# HgS Inhibits Oxidative Stress Caused by Hypoxia through Regulation of 5-HT Metabolism Pathway

**DOI:** 10.3390/ijms20061364

**Published:** 2019-03-18

**Authors:** Qiangqiang He, Ji Ma, Praveen Kumar Kalavagunta, Liangliang Zhou, Junyi Zhu, Jing Dong, Owais Ahmad, Yuzhi Du, Lixin Wei, Jing Shang

**Affiliations:** 1 Qinghai Key Laboratory of Tibetan Medicine Pharmacology and Safety Evaluation, Northwest Institute of Plateau Biology, Chinese Academy of Sciences, Xining, Qinghai 810008, China; 8075874@163.com (Q.H.); yzdu@nwipb.cas.cn (Y.D.); 2 University of Chinese Academy of Sciences, Beijing 100049, China; 3 State Key Laboratory of Natural Medicines, China Pharmaceutical University, Nanjing 210009, China; matthewmj@163.com (J.M.); kgpk81@gmail.com (P.K.K.); zhoulliang@163.com (L.Z.); rainbow811@foxmail.com (J.Z.); dongjingtx@gmail.com (J.D.); 4 Jiangsu Key Laboratory of TCM Evaluation and Translational Research, China Pharmaceutical University, Nanjing 211198, China; 5 School of Traditional Chinese Pharmacy, China Pharmaceutical University, Nanjing 211198, China; 6 School of Life Sciences, China Pharmaceutical University, Nanjing 211198, China; owaisk90@outlook.com

**Keywords:** hypoxia, reactive oxygen species, oxidative stress, 5-Hydroxytryptamine, cinnabar

## Abstract

This study aims to reveal the potential relationship between 5-HT and oxidative stress in the organism. Our in vitro experiments in RIN-14B cells showed that anoxia leads the cells to the state of oxidative stress. Administration of exogenous 5-HT exacerbated this effect, whereas the inhibition of Tph1, LP533401 alleviated the oxidative stress. Several research articles reported that Cinnabar (consists of more than 96% mercury sulfide, HgS), which is widely used in both Chinese and Indian traditional medicine prescriptions, has been involved in the regulation of 5-HT. The present research revealed that HgS relieved the level of oxidative stress of RIN-14B cells. This pharmacological activity was also observed in the prescription drug Zuotai, in which HgS accounts for 54.5%, and these effects were found to be similar to LP533401, an experimental drug to treat pulmonary hypertension. Further, our in vivo experiments revealed that the administration of cinnabar or prescription drug Zuotai in zebrafish reduced the reactive oxygen species (ROS) induced by hypoxia and cured behavioral abnormalities. Taken together, in organisms with hypoxia induced oxidative stress 5-HT levels were found to be abnormally elevated, indicating that 5-HT could regulate oxidative stress, and the decrease in the 5-HT levels, behavioral abnormalities after treatment with cinnabar and Zuotai, we may conclude that the therapeutic and pharmacologic effect of cinnabar and Zuotai may be based on the regulation of 5-HT metabolism and relief of oxidative stress. Even though they aren’t toxic at the present dosage in both cell lines and zebrafish, their dose dependent toxicities are yet to be evaluated.

## 1. Introduction

More than 140 million people live at altitudes higher than 2500 m on a global scale [1], and the Qinghai-Tibet Plateau, the largest region with the highest altitude (the average elevation over 4000 m), is sometimes called the roof of the world. The population residing above 4000 m altitude has been suffering from chronic hypoxia for the long term. Altitude-related health problems are particularly conspicuous in China since nearly 80 million people live above 2500 m with more than 12 million residing on the Qinghai-Tibet Plateau alone [2].

Oxidative stress refers to a condition in which cellular antioxidant defenses are inadequate to completely detoxify the free radicals generated in the body, which may be due to either excessive production of ROS or loss of antioxidant defenses, or typically, both [3]. Under hypoxic environmental conditions, enhanced ROS production was observed in both in vitro and in vivo [4], and overproduction of ROS was identified to be a leading cause of oxidative stress [5,6,7]. Other studies found that the level of 5-Hydroxytryptamine (also known as serotonin, 5-HT) changed in the hypoxic environment [8,9], and the 5-HT [10] and monoamine oxidase A (MAO-A) [11] are the sources of oxidative stress, which are critical players in the pathogenesis of several human diseases [12,13,14]. Recent research supports that metabolism pathway or the production of 5-HT may influence the ROS level. On the one hand, high altitude hypoxia was found to be involved in the development of diseases such as inflammatory bowel disease (IBD) [15,16], which is believed to be developed by increased 5-HT levels [17]. On the other hand, a recent study showed that the incidence of diseases such as depression, which is usually considered to be induced by the decline of 5-HT levels [18], were high in populations living at high altitudes [19].

Cinnabar, which consists of more than 96% mercury sulfide (HgS), has been used over 2000 years in traditional Chinese [20] and Indian (Ayurveda) medicines [21,22]. Its therapeutic effects were validated and confirmed in clinical applications [23,24]. In particular, we noticed the reports about the antioxidant [25] and 5-HT regulation properties [26] of cinnabar. On the other hand, cinnabar can be absorbed at high doses of oral administration, accumulates in the brain and other tissues, and causes mercury intoxication [27], while organomercurates and mercury and mercury vapor (Hg) possess strong toxicity on the central nervous system [28] mercuric sulfide (HgS) exhibits a dose-dependent toxicity in the doses ranging from 0.1 to 1 g/(kg day) but no ototoxic effect at 0.01 g/(kg day) [29]. Provided a toxicological basis for cinnabar-induced neurotoxic and ototoxic effects in offspring mice, changes in NOx levels and Na^+^/K^+^-ATPase activities were considered to be the underlying mechanism of the toxicological effects of cinnabar [30].

On the other hand, “Zuotai” is a traditional Chinese medicine that is used to treat stroke, brain trauma, neuroinflammation, and chronic ailments [31] is complex chemical composition containing 54.5% HgS [32]. Preparation process of Zuotai is the basis for its efficacy and toxicity [20]. Like other mercury-containing preparations, its application has always been controversial. It was pointed out that Zuotai induced apoptosis of hepatocytes by increasing cleaved-Caspase-3 production and decreasing the Bcl-2/Bax protein expression levels. Further, it is also known to induce hepatotoxicity and decreased CYP1A1, CYP1B1, and MT-1 mRNA levels in zebrafish [33].

Due to potential applications of these mercury based traditional Chinese medicines and insufficient data about their toxicities, a comprehensive research, which could provide convincing evidence for their application by evaluating the toxic effect of cinnabar and its formulations are pending to be carried out.

From the above reports it is clear that Zuotai in which major content is Hgs (54.5%) have 5-HT and ROS regulation properties and cinnabar is chiefly made up of Hgs (>96%), but is used in combination with other drugs. We hypothesized that the 5-HT and ROS regulation properties of Zuotai is chiefly due to HgS content in it. To prove our hypothesis, we are studying the 5-HT and ROS regulation properties of both Zuotai and cinnabar. If these properties of both Zuotai and cinnabar are comparable, we can assume that our hypothesis is proved.

## 2. Results

### 2.1. Relative ROS of RIN-14B Cells after Hypoxia Treatment

**Figure 1:**
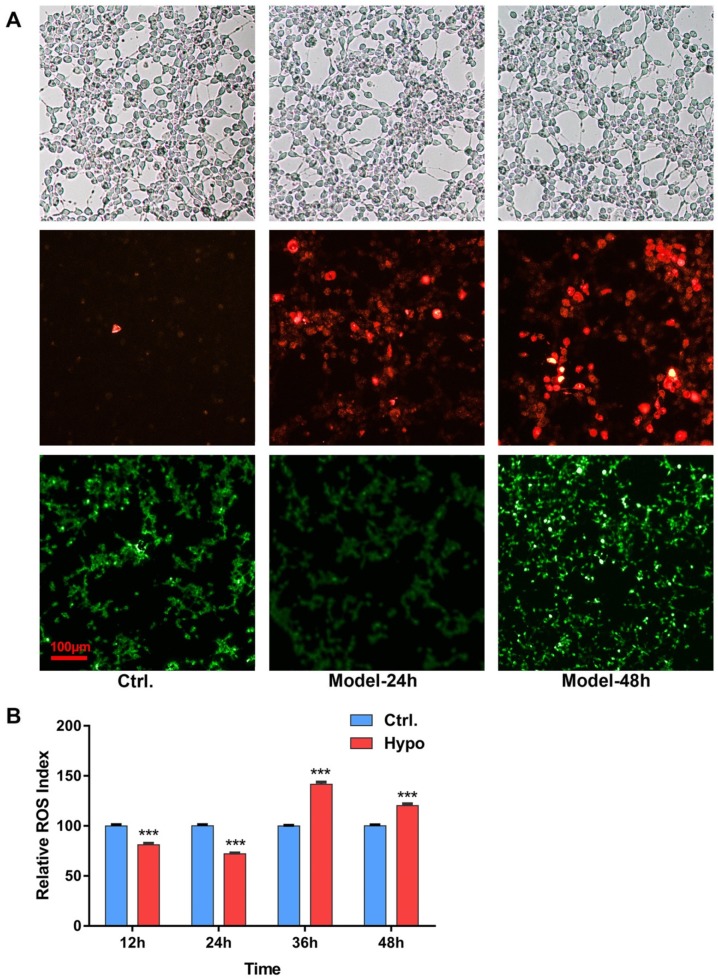
Relative ROS levels of RIN-14B Cells after hypoxia (5% O_2_) treatment. (**A**) Cells were stained with, Dihydroethidium (DHE), DCFH-DA, and were observed under a fluorescence microscope. Representative fluorescence images showed the ROS levels in RIN-14B cells compared to control after exposure to 5% O_2_ for 24 h/48 h. (Scale bar: 100 μm). (**B**) ROS were detected every 12 h with ROS assay kit; every group set the normoxic (21% O_2_) group as a control, (*n* = 8 each group). *** *P* < 0.001 represent compared with each control.

Two different durations of modeling treatments were set to investigate the ROS generation in RIN-14B cells, From light field images we can see, the density and morphology of cell growth in different treated groups did not show obvious difference; the results showed, after 24 h hypoxia (5% O_2_) treatments, the red fluorescence, which reflected the content of the superoxide anion, increased gradually with time within 48 h, the green fluorescence represented total ROS levels, did not increase compared to the control group at the first 24 h, but increased sharply when the modeling condition lasted to 48 h (Figure 1A). In the ROS assay kit experiment, within 48 h of hypoxia exposure, the ROS generation in RIN-14B cells decreased significantly over the normoxia control, from 12 h till 24 h. However, ROS increased from 36 h, and kept a constantly high level, the result was consistent with the result from 2,7-dichlorofluorescein diacetate (DCFH-DA) staining images (Figure 1B). 5% O_2_ duration for 48 h was chosen for further study.

### 2.2. The Effects of HgS and Zuotai on Abnormal ROS Level of RIN-14B Cells

**Figure 2:**
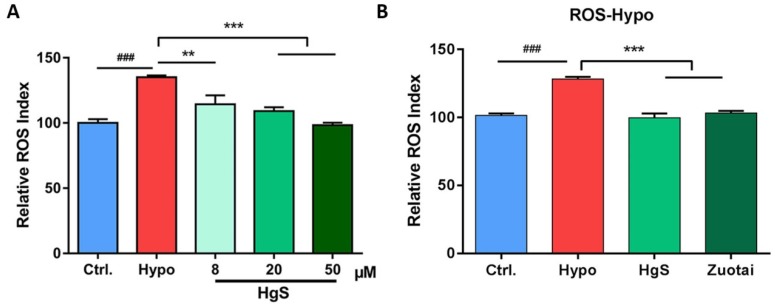
The effects of HgS and Zuotai to abnormal ROS level of RIN-14B cells. (**A**) Different doses of HgS inhibited the ROS induced by hypoxia modeling (5% O_2_, 48 h), (**B**) the effects of HgS and Zuotai on ROS inhibition. ^###^ *P* < 0.001 represent compared with control. ** *P* < 0.01, *** *P* < 0.001 represent compared with model (hypoxia). *P* < 0.05 was considered as statistically significant calculated by ANOVA.

As described before, we assume that the drugs which could regulate 5-HT may possess the effects to influence the level of ROS. In an in vitro study, three doses of HgS, 8, 20, and 50 μM were pre-administered to RIN-14B cells, the ROS was decreased with an increase in HgS concentration, presented in a concentration-dependent manner, and showed significant difference at 20 and 50 μM (Figure 2A). Thus, 20 μM was chosen for further research. To test if the ROS inhibition effects also exert in prescription Zuotai, the dose chosen has an equivalent weight of HgS, after pre-administration and exposure 48 h in 5% O_2_, both HgS and prescription Zuotai showed the activity of decreasing the ROS of RIN-14B cells (Figure 2B).

### 2.3. 5-HT Metabolism Pathway mRNA Expression in the RIN-14B

**Figure 3:**
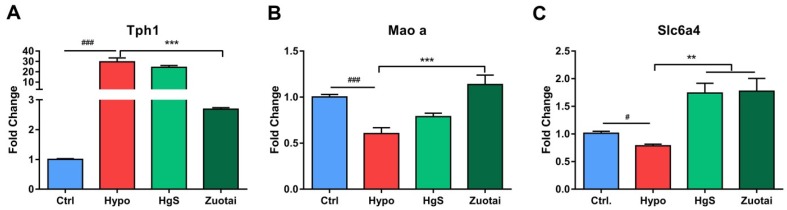
Effect of HgS/Zoutai on the metabolism of 5-HT pathway. (**A**) Tph1, (**B**) Maoa and (**C**) Slc6a4 mRNA expression of RIN-14B cells in different groups. (*n* = 6 each group). ^###^ *P* < 0.001 represent compared with control. ** *P* < 0.01, *** *P* < 0.001 represent compared with model (hypoxia). *P* < 0.05 was considered as statistically significant, calculated by ANOVA.

RIN-14B cells in different treated groups were harvested and RT-PCR experiments were performed to investigate the mRNA expressions of the metabolism of 5-HT pathway-related genes, Tph1, Maoa, and Slc6a4. The specific sequences of primers used in this study were synthesized by General Biotech Co., Ltd. (Shang Hai, China) and were shown in Table 1. The results showed the expression of Tph1 expression increased by 30-fold compared to the control in the model group (Figure 3A), HgS did not show potent activity to alleviate this change, but Zuotai decreased it significantly. The proteins regulated by Maoa and Sla6a4 play critical roles in the catabolism of 5-HT, hypoxia treatment induce down-regulation of Maoa and Sla6a4, Zuotai administration increased both genes expression significantly compared to Hypo group (Figure 3B,C), and HgS only rescued the Slc6a4 (Figure 3C).

### 2.4. The 5-HT Metabolism Pathway Influenced the ROS Generation in RIN-14B Cells

**Figure 4:**
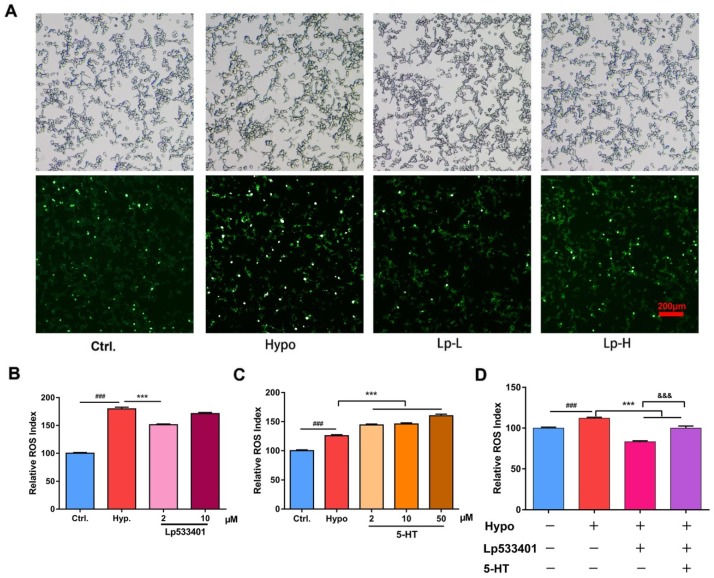
The 5-HT metabolism pathway influenced the ROS generation in RIN-14B cells. (**A**) Cells were stained with DCFH-DA and were observed under a fluorescence microscope. Representative fluorescence images showed the ROS level in RIN-14B cells compared to the control after exposure to 5% O_2_ for 48 h. (Lp-L: 2μM, Lp-H: 10 μM; Scale bar: 200 μm). ROS were detected with ROS assay kit after treated with (**B**) LP533401, (**C**) 5-HT, and (**D**) both. "+", the sample treated with the corresponding condition or the agents at certain concentrations, and “−”, untreated. (*n* = 6 each group). ^###^ *P* < 0.001 represent compared with control, *** *P* < 0.001 represent compared with model (hypoxia), ^&&&^ *P* < 0.001 represent compared with the LP533401 treated group. *P* < 0.05 was considered as statistically significant, calculated by ANOVA.

To confirm if the abnormal increase of 5-HT lead to overproduction of ROS, LP533401, the inhibition of Tph1 [34], and 5-hydroxytryptamine hydrochloride were administered to the RIN-14B cells, the results showed LP533401 decreased ROS under the concentration of 2 μM (Figure 4B), which consistent with the result of fluorescence images (Figure 4A). Exogenous 5-hydroxytryptamine hydrochloride increased ROS in a dose-dependent manner (Figure 4C). In addition, 5-hydroxytryptamine hydrochloride reversed the ROS level decreased by LP533401.

### 2.5. HgS and Zuotai Regulated Redox State of Larval Zebrafish

**Figure 5:**
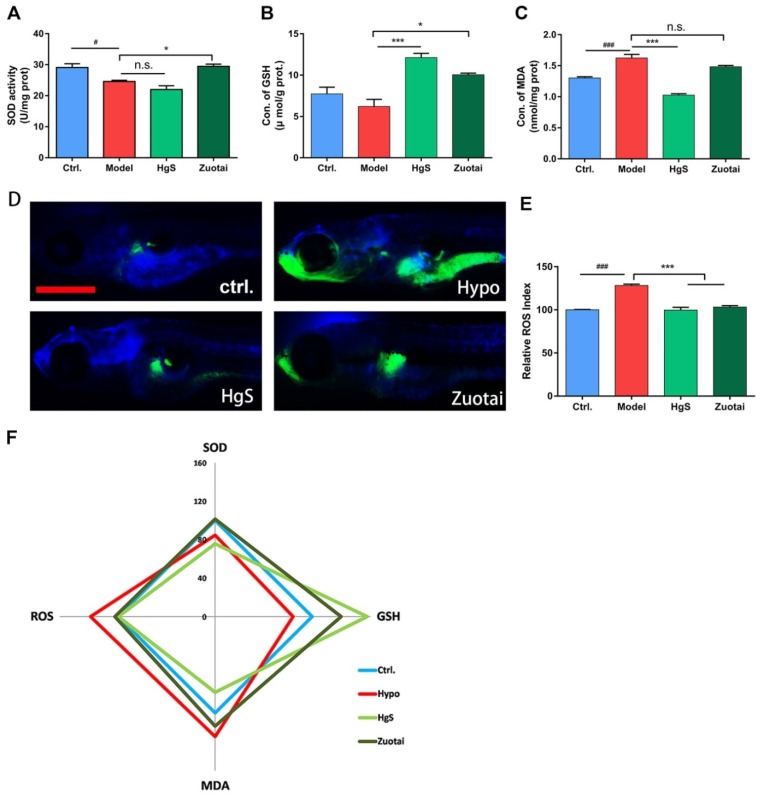
HgS and Zuotai regulated redox state of larval zebrafish. (**A**) SOD activity, (**B**) GSH content and (**C**) MDA content of each treated zebrafish tissues samples. (**D**) ROS production of larval zebrafish showed in the image (green fluorescence). (Scale bar: 2 mm). (**E**) Quantitation of relative ROS level of larval zebrafish. (**F**) Radar chart of 4 indexes showing the effects of HgS and Zuotai in vivo study. ^#^ *P* < 0.05, ^###^ *P* < 0.001 represent compared with control, * *P* < 0.05, *** *P* < 0.001 represent compared with the model (hypoxia group), n.s., no significant differences.

In vivo validation, 30 dpf larval zebrafish were administrated with HgS/Zuotai for two days (six times), and moved into the pre-treated hypoxic water for 6 h, it kept the dissolved oxygen in water decreased to 1.0–1.2 mg/L. After modeling, zebrafish was anesthetized in ice water mixture and determined the superoxide dismutase (SOD), glutathione (GSH), malonaldehyde (MDA), and ROS following the kits manufacturer’s instruction. In the model group, the activity of antioxidant enzyme SOD decreased significantly (Figure 5A), GSH also decreased compared to the control group, but had no significant differences (Figure 5B). The MDA increased (Figure 5C), indicating that the lipid may peroxide in hypoxia treatment. ROS increased consistency with the in vitro study; these abnormal changes were reversed by HgS/Zuotai either completely or partially.

### 2.6. Effects of HgS and Zuotai on Larval Zebrafish Behavior

**Figure 6:**
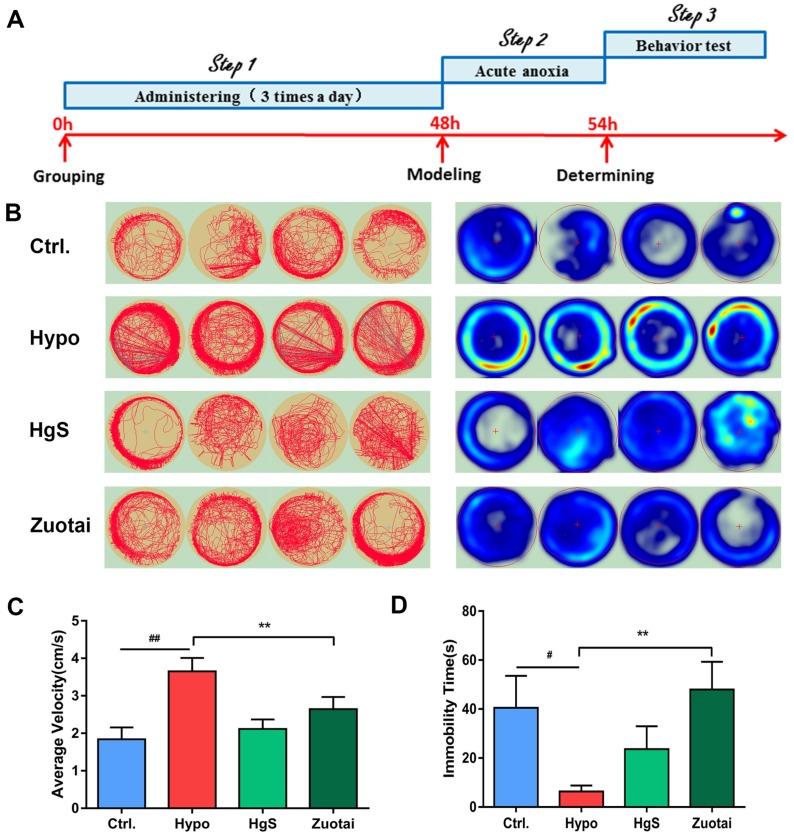
Effects of HgS and Zuotai on larval zebrafish behavior. (**A**) Schedule of the experiment. (**B**) Representative types of track plots and occupancy plots heatmap of larval zebrafish in 600 s. (*n* = 8 each group). (**C**) Average velocity and (**D**) the immobility time of larval zebrafish. ^#^ *P* < 0.05, ^##^ *P* < 0.01 represent compared with the control. ** *P* < 0.01 represent compared with the model (hypoxia group).

As previously described, 5-HT is an important neurotransmitter and has apparent neurotrophic activity; a praxeology experiment was set to determine the central nervous system activity of HgS and Zuotai. The results showed, under hypoxia treatment, zebrafish moved more and had less immobility time compared to control group, time spent in the central area significantly decreased (Figure 6B), in Zuotai group, the average velocity decreased, and the immobility time increased, the HgS group has a similar trend, but has no significant differences compared to the hypoxia group (Figure 6C,D).

## 3. Discussion

The present studies demonstrate that hypoxia-induced the overproduction of ROS and development of oxidative stress; the LP533401, which inhibited the function of Tph1, could alleviate the oxidative stress. It may be evident that 5-HT is one of the critical regulators of the oxidative stress signaling pathway. We proved that cinnabar and Zuotai possess antioxidant activity and showed the effects of relieving the shifting of antioxidant enzymes and ROS overproduction, attenuates or prevents the oxidative stress associated with hypoxia and are comparable to LP533401 in in vitro experiments.

ROS generation is a tightly controlled process and has pivotal roles in pathophysiological signaling [35]. It has been well demonstrated that an increased oxygen supply results in increased production of mitochondrial ROS [4].

On the other hand, some researchers have reported that hypoxia leads to oxidative stress [36,37], which also results in ROS overproduction through the mitochondrial electron transport system [38]. While free radicals are generated during normal metabolism and are required as intermediates in many biological reactions, over a threshold level they may damage macromolecules leading to oxidative stress [39].

In hypoxia induced RIN-14B cells, during the first 24 h, compared to the control group, there is a little decrease in the in the ROS level of the model group (Figure 1B). The most probable cause is lower levels of available O_2_ reducing the provision of oxygen to the cell, which is required as a substrate for free radical production [40]. However, after 36 h of inducing hypoxia, the ROS levels were drastically increased (Figure 1B). The reason behind the reversal of ROS levels may due to decreased oxygen availability to transfer electrons to terminal acceptors like oxidative phosphorylation, which leads to the accumulation of reducing equivalents in mitochondria. As a consequence, the reducing environment favors incomplete reduction of oxygen to highly reactive superoxide, peroxide, and hydroxyl radicals [41]. Additionally, other potential sources of ROS generation due to hypoxia, like increased activation of xanthine oxidase and phospholipase A2, etc., increases the release of oxygen radicals [42].

Cinnabar is also used as a sedative for its neuroactivity [29]. An Ayurvedic drug rasasindur, which contains HgS shows anti-oxidant property, has been noticed [25], our result confirmed this viewpoint. This result implied that the anti-oxidant property of the Ayurvedic drug rasasindur, as well as the prescription Zuotai, may due to the presence of HgS in their composition (Figure 2A,B).

Cinnabar is known to decrease 5-HT levels, but the exact mechanism is unknown [43]. To identify the possible mechanism, we focused on several critical factors of the 5-HT metabolism pathway [44] and the RT-PCR experiment proved that cinnabar, as well as the prescription Zuotai, influenced the mRNA levels of rate-limiting enzyme Tph1, Maoa, and Slc6a4 (Figure 3A–C). Remarkably, if Tph1 is taken as an index of anabolism, Maoa, and Slc6a4 should be indexes of catabolism. In the hypoxia group, Tph1 increased, while Maoa and Slc6a4 decreased; this may be the reason for the accumulated 5-HT under hypoxic conditions. HgS and Zuotai attenuated these abnormalities to varying extents (Figure 3A–C).

LP533401 is an experimental drug to inhibit the rate-limiting enzyme tryptophan hydroxylase 1 (Tph1). Mice repeatedly treated with LP533401 exhibited marked decrease in 5-HT levels in the gut, lungs, and blood [45]. In our result, LP533401 administration decreased the ROS level (Figure 4A,B), indicating lower 5-HT level results in lower levels of ROS. As Lp-H (10 μM) did not reduce as potent as Lp-L(2 μM), one possible reason may lie in the sensitivity and vulnerability enhancement in the hypoxia condition, and the physiological state of the RIN-14B cells would be influenced by the high concentration of LP533401 or dissolvent dimethyl sulphoxide. Exogenous 5-hydroxytryptamine hydrochloride increased the ROS directly and reserved the downward trend of after LP533401 administration (Figure 4C,D), indicating the regulatory effect of 5-HT to the oxidative stress.

The enzyme superoxide dismutase (SOD) is an antioxidant enzyme and acts as a primary defense system against the potentially destructive reactions catalyzed by O_2_ [46]. On the other hand, glutathione (GSH) is a tripeptide composed of g-glutamate, cysteine, and glycine, GSH plays a vital role in cellular defenses against oxidative damage [47]. In one word, antioxidant enzymes serve as the first line of defense toward scavenging ROS, and MDA is the marker of lipid peroxidation. In this study, SOD, as well as GSH, declined in model groups (Figure 5A,B), while MDA enhanced at the same condition (Figure 5C), these results are consistent with reported results [48], means redox status altered and there is an imbalance between pro-oxidant and antioxidant, the production of ROS confirmed it, and HgS and Zoutai administration reversed this trend partially or entirely, and shows the antioxidant activities.

Zebrafish, as a new model organism, has been gradually accepted and widely used in biology research due to its unique characteristics [49], a growing number of studies have exploited to examine the oxidative stress [50] and the role of hypoxia in brain dysfunction [51]. Here, we chose zebrafish as an animal model, attempted to understand the 5-HT in central never system, and examined the influence of hypoxia on the exploratory profiles of the zebrafish. After the 6 h hypoxia exposure, the exploratory activity of the model group enhanced significantly to their corresponding controls, indicating the level of 5-HT in central nerve system may also raise with the hypoxia treatment, while the parameters of HgS and Zuotai groups close to the control group (Figure 6). This result supported the assertion of neuroactivity of HgS.

5-HT has been demonstrated to be involved in many physiological and pathological processes. The metabolic pathways of 5-HT were clearly identified in the existing literature [44]. Many drugs also play an active role by acting on the receptors of 5-HT or 5-HT metabolic pathway. However, in addition to the functions widely understood and well known. This research shows that mineral medicine HgS, which has been used to treat insomnia, mental excitement, and gastrointestinal diseases in traditional Chinese medicine, also plays a regulatory role on the oxidative stress state of organism through the regulation 5-HT metabolism, this may be the reason why oxidative stress in the model can be improved by reducing the level of 5-HT. The results were confirmed in vitro and in vivo experiments with RIN-14B cells and zebrafish as a model, respectively.

**Figure 7:**
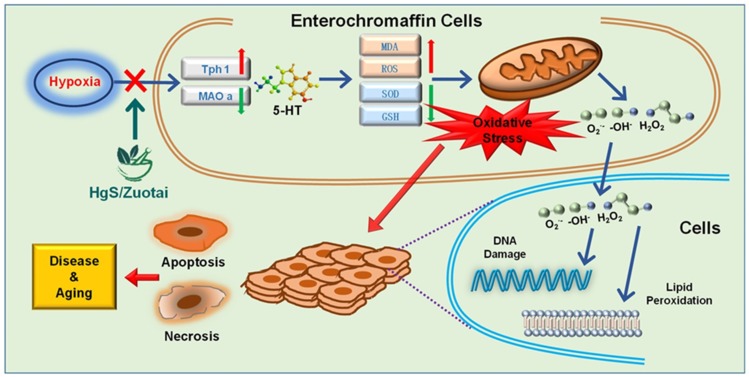
The proposed mechanism of HgS and Zuotai on hypoxia-induced oxidative stress.

We further discuss the involvement of 5-HT in the oxidative stress after exposure to hyperoxia, by our present study, we propose the following assertion that HgS and Zuotai adjust the imbalance of Pro-oxidant and antioxidant, provides a protective effect in oxidative stress. (Figure 7).

## 4. Materials and Methods

### 4.1. Chemicals and Solution

Hydrogen peroxide solution (3wt % in H_2_O) and 2′,7′-dichlorofluorescein diacetate (DCFH-DA) was purchased from Sigma Aldrich (St. Louis, MO, USA), HgS was purchased from Alfa Aesar (Ward Hill, MA, USA), Zuotai was provided by the Northwest Plateau Institute of Biology of Chinese Academy of Sciences. LP533401 was purchased from Sigma Aldrich (St. Louis, MO, USA).

RPMI medium 1640 was purchased from Gibco Life Technologies (Grand Island, NY, USA), FBS was purchased from Hyclone (Victoria, Australia), serotonin hydrochloride, and DMSO (HPLC grade) were purchased from Sigma Aldrich (St. Louis, MO, USA). NaHCO_3_ was obtained from Sinopharm Chemical Reagent Co. Ltd. (Shanghai, China).

SOD, GSH, MDA, and ROS were measured by commercial assay kits (Nanjing Jiancheng Bioengineering Institute, Nanjing, China) following the manufacturer’s instructions.

### 4.2. Materials

RIN-14B cell line was purchased from the American Type Culture Collection (ATCC; CRL-2059). The wild-type AB-line zebrafish were obtained from China Zebrafish Resource Center (Wuhan, China).

### 4.3. Cell Culture and Treatments

RIN-14B cells were recovered, cultured, passaged to the third generation. These cells were cultured in RPMI 1640 medium containing 10% fetal bovine serum (FBS) in an incubator (Thermo Fisher Scientific Inc. Marietta, OH, USA) with 5% CO_2_ and at 37 °C for 20 h for cells attachment, then the medium was replaced with FBS free 1640 medium and transferred to an anoxic incubator (Thermo Fisher Scientific Inc. Marietta, OH, USA) in which 5% O_2_ sustained to conduct 48 h modeling stage.

### 4.4. Biochemical Measurement

The superoxide dismutase (SOD) activity, Glutathione (GSH) activity and malondialdehyde (MDA) levels of cells and larval zebrafish samples were measured by commercial assay kits (Jiancheng, Nanjing, China) following the manufacturer’s instructions.

Reactive Oxygen Species Kit (Beyotime Biotechnology, China) was used for the quantitation of intracellular ROS in cell and larval zebrafish samples by following the manufacturer’s instructions.

SOD and GSH was determined with an ultraviolet spectrophotometer (TU-1810, PERSEE, Beijing China) at wavelength 405 nm and 450 nm, respectively, and MDA was measured with a microplate reader of the multi-wavelength measurement system (Synergy HT, Bio-Tek. Winooski, VT, USA) at wavelength 532 nm.

### 4.5. Measurement of ROS Generation

DHE (Dihydroethidium) assay kit was obtained from beyotime (Shanghai, China), The fluorescent probe for detecting intracellular superoxide anion levels used commonly. The intracellular generation of ROS is an index of oxidative stress. As an indicator of ROS, 2,7-dichlorofluorescein diacetate (DCFH-DA) can cross the cell membrane and enters the cell. The ROS inside the cell oxidize DCFH-DA to dichlorofluorescein (DCF), which cannot penetrate the cell membrane, and thus, can be detected by its green autofluorescence (excitation 485 nm, emission 525 nm).

RIN-14B cells (1 × 10^5^ cells/mL) were inoculated to 96-well black plates with 0.2 mL per well and incubated for 24 h or until it reaches about 80% confluency, treated with HgS or Zuotai and incubated in an anoxic incubator for 48 h. The cells in different treatment conditions were incubated with 10 μM DCFH-DA at 37 °C for 30 min. The medium was aspirated carefully and 100 μL PBS was added into each well to wash the DCFH-DA adsorbed on the surface of cells. ROS generation was determined by fluorescence intensity with the setting of 485 and 525 nm for excitation and emission wavelengths.

### 4.6. Maintenance of Zebrafish and Treatments

The adult zebrafish maintained in circulating water at 28 °C with a 14:10 h light-dark cycle, culture density was 8 tail/2.5 L/tank. Embryos were generated by natural spawning and were allowed to grow for the next 30 days in a light incubator.

**Figure 8:**
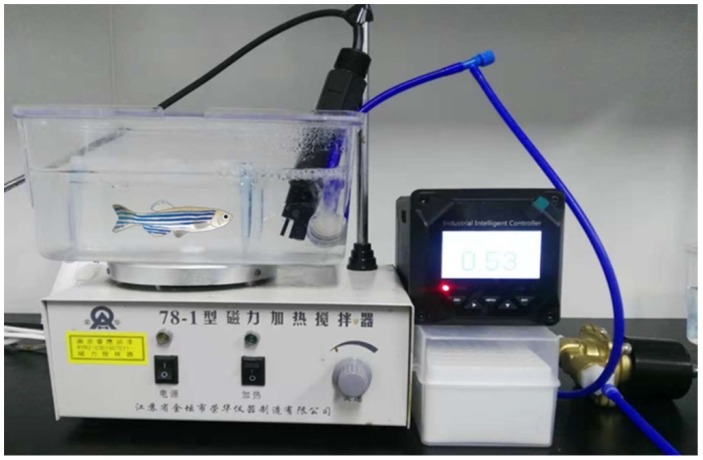
The experimental device of zebrafish hypoxia model.

HgS and Zuotai were mixed with starter diet directly (1:5, *w*/*w*). The fine particles of HgS/Zuotai can be mixed uniformly for the forage is loose and porous, this property may be helpful to form homogeneous and stable drug delivery system. On the 31st day, the larval zebrafish were grouped, there were four groups, control group, model group, HgS treated group and Zuotai treated group, and 10 tails each group with 1.5 L water in one tank. Larval zebrafish were pretreated with drug-forage mixture three times per day (0.3 g/10 larval zebrafish), administration for two days. Then, three tanks except control group were closed to initiate the hypoxia experiments. The oxygen content of the water was reduced from 6.5 ± 0.5 mg/L to 0.8–1.0 mg/L by bubbling nitrogen gas (99.999% purity), and hypoxic condition was maintained for 6 h. The larval zebrafish were carefully transferred into tanks with 1 L water with 0.8–1.0 mg/L oxygen content water, the concentration of oxygen in the tank was monitored and supplemented nitrogen in real time to remain constant. We developed a device, by which we can control the concentration of oxygen accurately (Figure 8). The hypoxic course duration was for 6 h, and then behaviors of larval zebrafish were examined and recorded for 12 min.

The animal experiments were carried out by following Jiangsu Provincial Standard Ethical Guidelines for the use of experimental animals and were approved by the Science and Technology Department of Jiangsu Province.

### 4.7. Fluorescence Photography

For RIN-14B cells, for taking green fluorescence photography with the ROS kit, cells were incubated with 15 mM DCFH-DA work solution for 30 min at 37 °C, washed thrice with PBS and photographed with a fluorescence microscope (Nikon Eclipse Ni-U) within 15 min.

When it comes to orange fluorescence photography with DHE kit, cells were incubated with 20 mM DHE work solution for 30 min at 37 °C, washed twice with PBS and photographed with a fluorescence microscope (Nikon Eclipse Ni-U) within 15 min.

For larval zebrafish, after incubation with 15 μM DCFH-DA work solution (diluted with embryo water) for 30 min at 27 °C, 6 larval were transferred into 200 mL embryo water for 3 min to wash the DCFH-DA physically absorbed on the body surface, then stained cutaneous cells with DAPI (Sigma, USA) to highlight the green fluorescence from the cells in which ROS were accumulated. Larval zebrafish were kept into the 3.5% sodium carboxymethylcellulose (CMC-Na) on a glass slide for fixation. The images were captured immediately using a fluorescence stereoscope (Nikon Eclipse Ni-U), and all the captures were taken in the dark room with the same parameters (exposure time, ISO and aperture) for comparison with different groups.

### 4.8. Quantitative Real-Time PCR Studies

RNA was extracted from the harvested RIN-14B cells and reverse transcribed using commercial kits (Takara, Japan) according to the manufacturer’s protocol. The primers sequences were listed in Table 1.

Real-time PCR was performed using a 96-well standard block (Applied Biosystems. Blk33, Marsiling Industrial Estate 33#07-06, Singapore) and StepOnePlus Real-Time PCR System instrument (Applied Biosystems. Blk33, Marsiling Industrial Estate 33#07-06, Singapor) in 20 μL reaction mixtures. Each sample was loaded in triplicate; amplification of GADPH was used as an internal reference gene. PCR amplifications were performed 40 cycles with each cycle at 95 °C for 5 s and 60 °C for 30 s. Expression levels of each targeting mRNAs normalized to GAPDH, and the relative amount of RNA was quantified using the comparative cycle threshold (CT) (2^−ΔΔ*C*t^) method.

### 4.9. Statistical Analysis

All the data were presented in this study were expressed as mean ± SEM. Statistical analysis for the treatment groups and corresponding controls were carried out by one-way analysis of variance (ANOVA) for the significant difference. The differences between groups were considered statistically significant for *P*-value < 0.05. All the data calculated by Graph Pad PRISM (GraphPad Prism 7.00, San Diego, CA, USA).

## 5. Conclusions

In conclusion, the present study elucidated the underlying internal connection between 5-HT and oxidative stress; confirmed that hypoxia could induce oxidative stress; and proved the 5-HT might be one of the effective regulators of the oxidative stress signaling pathway for the first time. HgS possess antioxidant activity and can relieve the shifting of antioxidant enzymes and ROS overproduction under anoxic condition, attenuate or prevent the oxidative stress induced by hypoxia, may be due to its effect on 5-HT metabolism regulation. At the present experimental dosage, both of HgS and Zuotai, what was used here was not found to show any toxicity in both RIN-14B cell lines and zebrafish, which means HgS can be of therapeutic use. However, further studies are necessary to explore the dose dependent toxicity.

The 5-HT and ROS regulatory efficiencies of HgS and Zuotai are comparable and seems to follow the pathway similar to the Tph1 inhibitor LP533401. Further, this study provided necessary information to understand the pharmacologic effect and mechanism of action of HgS and prescription drug Zuotai on maintaining redox status. These results are supporting our hypothesis that the 5-HT pathway might represent a potential therapeutic target for the treatment of oxidative stress-related diseases for which Zuotai is under use, and drives research towards the development of drugs targeting factors in the 5-HT pathway to treat such diseases.

## Figures and Tables

**Table 1:** Primers used for the detection of RIN-14B for RT-PCR reactions.

Gene Name	Forward Primer Sequence	Reverse Primer Sequence
Gapdh	TCTCTGCTCCTCCCTGTTC	ACACCGACCTTCACCATCT
Tph1	GTCCCTCTCTTGGCTGAA	TGAACCGTCTCCTCTGAA
Maoa	AAGACACGCTCAGGAATG	TGAACCGTCTCCTCTGAA
Slc6a4(SERT)	AGCGATGTGAAGGAGATGCT	GGACGACATCCCTATGCAGT

## Abbreviations

ROS	reactive oxygen species
5-HT	5-hydroxytryptamine
IBD	inflammatory bowel disease
FBS	fetal bovine serum
SOD	superoxide dismutase
GSH	glutathione
MDA	malondialdehyde
Tph1	Tryptophan hydroxylase-1
Maoa	monoamine oxidase A
Slc6a4	Solute Carrier Family 6 Member 4
Ctrl.	control
Hypo	hypoxia group
